# Uric acid as a prognostic factor and critical marker of COVID-19

**DOI:** 10.1038/s41598-021-96983-4

**Published:** 2021-09-07

**Authors:** Gang Li, Xia Wu, Chen-liang Zhou, Ye-ming Wang, Bin Song, Xiao-bin Cheng, Qiu-fen Dong, Liu-lin Wang, Sha-sha You, Yuan-ming Ba

**Affiliations:** 1 Department of Emergency and Critical Care Medicine, Hubei Provincial Hospital of Traditional Chinese Medicine, Wuhan, 430061 China; 2 Hubei Province Academy of Traditional Chinese Medicine, Wuhan, China; 3 Department of Respiration, Hubei Provincial Hospital of Traditional Chinese Medicine, Wuhan, China; 4 Department of Critical Care Medicine, Renmin Hospital of Wuhan University, Wuhan, China; 5 Department of Critical Care Medicine, Hubei Provincial Hospital of Integrated Chinese & Western Medicine, Wuhan, China; 6 Department of Critical Care Medicine, Jin Yin-Tan Hospital, Wuhan, China; 7 Department of Nephrology, Hubei Provincial Hospital of Traditional Chinese Medicine, Wuhan, China

**Keywords:** Predictive markers, Viral infection

## Abstract

The purpose of this study is to explore whether uric acid (UA) can independently act as a prognostic factor and critical marker of the 2019 novel corona virus disease (COVID-19). A multicenter, retrospective, and observational study including 540 patients with confirmed COVID-19 was carried out at four designated hospitals in Wuhan. Demographic, clinical, laboratory data were collected and analyzed. The primary end point was in-hospital death of patients with COVID-19. The concentration of admission UA (*ad*UA) and the lowest concentration of uric acid during hospitalization (*low*UA) in the dead patients were significantly lower than those in the survivors. Multivariate logistic regression analysis showed the concentration of *low*UA (OR 0.986, 95% CI 0.980–0.992, *p* < 0.001) was able to independently predict the risk of in-hospital death. The mean survival time in the low-level group of *low*UA was significantly lower than other groups. When *low*UA was ≤ 166 µmol/L, the sensitivity and specificity in predicting hospital short-term mortality were 76.9%, (95% CI 68.5–85.1%) and 74.9% (95% CI 70.3–78.9%). This retrospective study determined that the lowest concentration of UA during hospitalization can be used as a prognostic indicator and a marker of disease severity in severe patients with COVID-19.

## Introduction

The 2019 novel corona virus disease (COVID-19) is a newly emerging disease. It is highly infectious and has spread to most parts of the world^1–3^. By May 11, 2021, over 150 million people have been infected worldwide and more than 3 million people have died from the disease^4^. Sang You et al. showed that the mortality rate of critically ill patients was as high as 60%^5^. Since it was declared a global pandemic, COVID-19 has ravaged many countries around the world, overwhelming many medical systems. The WHO currently estimates the global mortality of COVID-19 is 2.2%^6^. Meanwhile, our understanding of the COVID-19 clinical characteristics is increasing, including the presence of inflammatory cytokine storm and mucus blockage of alveoli and small airway^6–8^. At present, there are drugs showing a certain therapeutic potential, including antiviral drugs, anti-SARS-CoV-2 monoclonal antibodies, anti-inflammatory drugs, immunomodulators^9–11^. These drugs have shown a certain role in the treatment of COVID-19.

Individuals of all ages are at risk of contracting COVID-19 and at risk of serious consequences. However, the risk of severe COVID-19 infection is increased in elderly patients over 60 years old and with diseases or underlying medical risks (obesity, cardiovascular disease, chronic kidney disease, diabetes, chronic lung disease, smoking, cancer)^12^. The American College of Cardiology issued a clinical bulletin in March 2020, which reported that the mortality of patients with previous diseases was higher than that of patients without previous diseases^13^. Compared with diabetes mellitus (7.3%), chronic obstructive pulmonary disease (6.3%), hypertension (6.0%) and cancer (5.6%), COVID-19 patients with cardiovascular disease had the highest mortality (10.5%). In contrast, patients without previous disease had a fatality rate of < 1%^13^. Some laboratory parameters can predict the prognosis of covid-19. Findings commonly associated with poor prognosis include elevated C-reactive protein (CRP), LDH, d-dimer levels, and high-sensitivity cardiac troponin I^14^. However, the role of these and other biomarkers in the pathogenesis of SARS CoV-2 remains to be confirmed.

Uric acid (UA) is a catabolic product of purine, which comes from RNA and DNA. As a metabolic index, it is less affected by other factors except drugs and high-purine diet. Previous studies have shown that UA is closely related to the activation of the immune system and scavenging of oxygen free radicals^15–17^. Wu et al. found that hypouricemia is not rare in patients with SARS-CoV infection and may reflect the severity of disease and predict poor patient outcomes^18^. However, only 60 SARS patients were included in this study, and UA was not collected on a fixed schedule. 12,413 patients with COVID-19 were included in Liu's retrospective study. They found that low levels of uric acid on admission were associated with 28 day all-cause mortality in COVID-19 patients^19^. This study did not dynamically observe changes in uric acid levels, and did not analyze severe patients alone. Therefore, the purpose of this study is to explore whether UA can independently predict the prognosis of severe patients with COVID-19 and whether it can be used as an index to evaluate the degree of the disease.

## Methods

### Study design and participants

This is a multicenter, retrospective, and observational study. The retrospective analysis was carried out at four designated hospitals for COVID-19, including Hubei Provincial Hospital of Traditional Chinese Medicine, Renmin Hospital of Wuhan University, Hubei Provincial Hospital of Integrated Chinese & Western Medicine and Jin Yin-tan Hospital, Wuhan. The risk of death in patients with mild COVID-19 was very low, and therefore, confirmed patients with a severe and critical disease status were included. Severely ill patients were included in the study if they met any of the following criteria: (1) respiratory distress and respiratory rate (RR) was ≥ 30 times/min; (2) oxygen saturation in a resting state was ≤ 93%; and (3) arterial partial pressure of oxygen (PaO_2_)/fraction of inspired oxygen (FiO_2_) was ≤ 300 mmHg^20^. Critically ill patients were included if they met any of the following criteria: (1) respiratory failure and need for mechanical ventilation; (2) shock; and (3) other organ failure requiring intensive care unit (ICU) monitoring^20^. Positive results for the real-time polymerase chain reaction testing of respiratory or blood samples were defined as confirmed cases^21^. Sepsis was identified according to the third international consensus definitions for sepsis and septic shock^22^. The accounting test was conducted by the local Centers for Disease Control or a qualified medical institution laboratory according to the guidance. The inclusion time was from January 2, 2020 to February 15, 2020 for discharged and dead patients. The severity of patients with COVID-19 was determined using the analysis of electronic medical records, nursing records, and related examinations. All data review was performed by experienced ICU doctors. Those with < 24 h of hospitalization, less than three routine blood examinations, blood biochemical and blood gas analyses during hospitalization, or oral administration of uric acid-lowering drugs 1 week before admission and during hospitalization were excluded. A total of 540 patients participated in the study, including 389 severe and 151 critically ill patients, with hospital death as the end event. Due to the speed of COVID-19 spread and the risk of infection, exemption from the written informed consent was obtained. This study was approved by the ethics committee of Hubei Provincial Hospital of Traditional Chinese Medicine (HBZY2020-C14-01). The ethics committee review boards that approved this study waived the need for informed consent. We confirmed that all methods were performed in accordance with the relevant guidelines and regulations of WHO guidelines and complied with the Declaration of Helsinki.

### Data collection

Data for age, gender, history of chronic diseases (hypertension, coronary heart disease, and diabetes), vital signs, laboratory values, changes in UA and absolute lymphocyte count (ALC) during hospitalization, hospitalization time, chest imaging characteristics, and prognosis of patients with COVID-19 were collected. Assuming that the drug treatment of patients was in accordance with the national diagnosis and treatment standards, patient drug information was not collected. In addition, the Glasgow Coma Scale, sequential organ failure assessment, acute physiology and chronic health assessment II scores, partial pressure of oxygen (PaO_2_) and lactate concentration, PaO_2_/FiO_2_, and other indicators for subsequent analysis data were collected for some patients in the ICU.

### Statistical analysis

Demographic and medical data meeting normal distribution requirements were presented as mean ± SD. Data with a skewed distribution were presented as median (quantile). Categorical variables were described as frequency rates and percentages. We assessed differences between survivors and non-survivors using two-sample *t* test or Mann–Whitney test depending on normal distribution or skewed distribution data for continuous variables and Chi-square test for categorical variables. After adjusting for age and sex, general linear model (GLM) was used to analyze the differences in the level of UA and ALC between survivors and non-survivors. Multivariate logistic regression was used to analyze the association between UA and ALC with a death risk in hospital. Survival curves between the tertiles of the lowest level of uric acid during hospitalization (*low*UA) were estimated according to the Kaplan–Meier method, and compared by the log-rank test. To determine the discriminative power of *low*UA and admission ALC (*ad*ALC) for the death in hospital, the area under the receiver operating characteristic (AROC) curves were calculated. All statistical analyses were performed using the SPSS 22.0 software (SPSS Inc, Chicago, Illinois) and *p* < 0.05 was considered to be statistically significant.

### Ethics approval and consent to participate

Exemption from the written informed consent was obtained. The study was approved by the ethics committee of Hubei Provincial Hospital of Traditional Chinese Medicine (HBZY2020-C14-01).

## Results

The average age for the 540 patients in this study was 54.6 ± 16.0 years old. There were 262 males (48.5%) and 120 deaths (22.2%). Patients who died were older, had a higher proportion of men, and a greater history of previous chronic disease. Compared to the survivors, the dead patients had a higher liver enzyme index [alanine aminotransferase (ALT), aspartate aminotransferase (AST)], renal function index [blood urea nitrogen (BUN), creatinine (Cr)], and fasting blood glucose level. The results of blood analysis showed that the white blood cell count of the dead patients was higher than that of the survivors, while the lymphocyte count, lymphocyte percentage and the lowest ALC (low ALC) during hospitalization were significantly lower than those of the survivors. In terms of the inflammatory factors, the levels of C-reactive protein (CRP) and procalcitonin (PCT) upon admission were significantly higher in the dead patients. There were no significant differences in interleukin (IL)-6 levels. TroponinI (TnI), which is an index that reflects myocardial injury, was also higher in the dead patients. There were no differences in brain natriuretic peptide (BNP) levels. The concentration of *ad*UA and *low*UA in the dead patients were significantly lower than those in the survivors (Table 1). General linear modeling (GLM) analysis showed that after adjusting for age and gender, the concentration of *ad*UA (non-survivors vs survivors: 218.7 vs 258.3 µmol/L, *p* < 0.001) and the level of *low*UA (non-survivors vs survivors: 123.6 vs 209.4 µmol/L, *p* < 0.001) in the dead patients were still significantly reduced. The level of uric acid in the dead patients was decreased to its lowest value on the fifth day after admission.

**Table 1:** Baseline characteristics of patients with severe COVID-19 pneumonia.

	Survivors (n = 420)	Non-survivors (n = 120)	All patients	*p* value	Normal range
Age (years)	50.4 ± 14.4	69.3 ± 12.1	54.6 ± 16.0	< 0.001	NA
Male n, (%)	190 (45.2%)	72 (60.0%)	262 (48.5%)	0.005	NA
Coronary artery disease n, (%)	27 (6.5%)	19 (15.8%)	46 (8.6%)	0.002	NA
Hypertension n, (%)	92 (22.0%)	52 (43.3%)	144 (26.8%)	< 0.001	NA
Diabetes mellitus n, (%)	66 (15.7%)	34 (28.3%)	100 (18.5%)	0.002	NA
Admission SBP (mmHg)	124.1 ± 15.5	132.4 ± 19.7	126.3 ± 17.1	< 0.001	< 140
Admission DBP (mmHg)	78.1 ± 10.4	78.7 ± 11.6	78.2 ± 10.7	0.631	< 90
ALT(U/L)	35.9 ± 33.1	46.3 ± 53.2	38.2 ± 38.7	0.010	9–50
AST (U/L)	30.5 ± 18.2	54.8 ± 59.2	36.0 ± 34.0	< 0.001	15–40
BUN (mmol/L)	4.68 ± 2.08	9.09 ± 6.82	5.64 ± 4.09	< 0.001	3.1–8.0
Cr (µmol/L)	67.0 ± 28.1	100.4 ± 72.5	74.2 ± 44.0	< 0.001	57–111
*ad*UA (µmol/L)	265.1 ± 97.7	237.3 ± 121.5	261.1 ± 103.6	0.010	155–428
*low*UA (µmol/L)	220.5 ± 77.5	137.6 ± 74.6	202.1 ± 84.2	< 0.001	155–428
WBC, × 10^9^/L	5.18 (3.94, 7.00)	7.80 (5.49, 11.53)	5.67 (4.16, 7.86)	< 0.001	3.5–9.5
*ad*ALC, × 10^9^/L	1.10 (0.77, 1.57)	0.63 (0.41, 0.93)	1.00 (0.66, 1.40)	< 0.001	1.1–3.2
*low*ALC, × 10^9^/L	1.02 (0.72, 1.32)	0.41 (0.26, 0.68)	0.89 (0.55,1 .24)	< 0.001	1.1–3.2
LYM%	20.29 (6.84, 29.09)	5.02 (1.61, 11.74)	16.4 (4.05, 27.6)	< 0.001	20–50
IL-6 (pg/mL)	7.72 (5.33, 15.47)	11.06 (7.84, 23.60)	9.18 (5.95, 20.61)	0.155	< 7.0
hs-CRP (mg/L)	34.3 ± 42.4	94.1 ± 64.0	48.1 ± 54.3	< 0.001	0–3
PCT (ng/mL)	0.05 (0.044, 0.090)	0.16 (0.08, 0.37)	0.055 (0.050, 0.143)	< 0.001	0–0.052
NT-proBNP (pg/mL)	62.5 (22.8, 144.7)	98.3 (48.7, 245.4)	69.3 (30.1, 186.0)	0.218	0–125.2
cTnI (pg/mL)	0.041 ± 0.252	0.230 ± 0.619	0.093 ± 0.395	< 0.001	0–0.06
Sepsis (n, %)	166 (39.5%)	97 (80.8%)	263 (48.7%)	< 0.001	NA
Hospitalization days, days	12.0 ± 6.8	9.5 ± 6.3	11.5 ± 6.7	< 0.001	NA
Days of hospital admission to *low*UA, days	4.0 ± 3.6	4.9 ± 4.5	4.2 ± 3.8	0.017	NA
Onset of symptom to admission hospital, days	11.8 ± 3.1	7.7 ± 3.2	10.5 ± 3.2	< 0.001	NA
Onset of symptom to *low*UA, days	15.8 ± 5.2	11.7 ± 4.3	13.8 ± 4.9	< 0.001	NA

*ad*ALC, admission absolute lymphocyte count; *low*ALC, the lowest level of ALC during hospitalization; ALT, alanine aminotransferase; AST, aspartate aminotransferase; BUN, blood urea nitrogen; Cr, creatinine; cTnI, cardiac troponin I; DBP, diastolic blood pressure; hs-CRP, high-sensitivity C reactive protein; IL-6, interleukin-6; LYM%, Lymphocyte percentage; NT-proBNP, N-Terminal pro-brain natriuretic peptide; PCT, procalcitonin; SBP, systolic blood pressure; *ad*UA, admission uric acid; *low*UA, the lowest level of uric acid during hospitalization; WBC, white blood cell.

As shown in Table 1, 263 (48.7%) patients with COVID-19 complicated sepsis. In all of patients, 80% of the dead patients had sepsis, while only 40% of the survival patients had sepsis (p < 0.001). In addition, the *low*UA in patients with sepsis was significantly lower than that in patients without sepsis (193.2 ± 90.2 vs 220.6 ± 80.6, p = 0.005). However, there was no difference in *ad*UA between the patients with sepsis and those without sepsis (249.3 ± 95.1 vs 258.9 ± 83.2 µmol/L, p = 0.388). Among the dead patients, the mean time from onset of symptoms to hospital admission was 8.0 ± 3.3 days, which tended to be shorter than for recovered patients (11.8 ± 3.0 days).The mean time form hospital admission to lowUA in dead patients was 5.1 ± 4.5 days. However, UA concentrations of the dead patients was faster than survival patients deceased to the lowest value after onset of symptom (12.8 ± 4.6 vs 15.6 ± 5.1 days, p < 0.001).

Correlation analysis showed a significant correlation between *ad*UA and *ad*ALC (r = 0.140, *p* = 0.001). There was also a significant correlation between *low*UA and *low*ALC (r = 0.330, *p* < 0.001). In addition, the level of *ad*UA was negatively correlated with WBC (r = − 0.111, *p* = 0.011) and CRP (r = − 0.247, *p* < 0.001). The *low*UA was also negatively correlated with WBC(r = − 0.223, *p* < 0.001) and CRP(r = − 0.401, *p* < 0.001). However, there was no significant correlation between PCT and *ad*UA and *low*UA.

Multivariate logistic regression analysis showed that in the absence of adjusted variables, both the concentration of *ad*UA and *low*UA could predict the risk of in-hospital death. However, after adjustment for age, gender, history of chronic diseases, WBC, hs-CRP, cTnI, and Cr, only the concentration of *low*UA (OR 0.986, 95% CI 0.980–0.992, *p* < 0.001, Table 2) was able to independently predict the risk of in-hospital death. Meanwhile, after adjustment for these variables, the value of *ad*ALC could not independently predict the risk of in-hospital death (OR 0.656, 95% CI 0.381–1.132, *p* = 0.130, Table 2). After adjusted variables, days of hospital admission to *low*UA could independently predict the risk of in-hospital death (OR 1.097, 95% CI 1.014–1.187, *p* = 0.022, Table 2).

**Table 2:** Logistic regression analysis for *ad*UA, *low*UA, *ad*ALC and days to *low*UA to predict the death of COVID-19 patients.

	β-Coefficient	SE	OR	95% CI	*p* value
***ad*UA**					
Unjusted	− 0.002	0.001	0.998	0.995–1.000	0.037
Model 1	− 0.005	0.001	0.995	0.993–0.998	< 0.001
Model 2	− 0.005	0.001	0.995	0.992–0.997	< 0.001
Model 3	− 0.005	0.002	0.995	0.991–1.002	0.071
***low*UA**					
Unjusted	− 0.018	0.002	0.982	0.978–0.986	< 0.001
Model 1	− 0.017	0.002	0.983	0.978–0.987	< 0.001
Model 2	− 0.018	0.002	0.982	0.978–0.987	< 0.001
Model 3	− 0.014	0.003	0.986	0.980–0.992	< 0.001
***ad*ALC**					
Unjusted	− 2.008	0.292	0.134	0.076–0.238	< 0.001
Model 1	− 1.229	0.325	0.293	0.155–0.553	< 0.001
Model 2	− 0.674	0.298	0.510	0.284–0.915	0.024
Model 3	− 0.421	0.278	0.656	0.381–1.132	0.130
**Days of hospital admission to *low*UA**					
Unjusted	0.059	0.025	1.061	1.010–1.115	0.020
Model 1	0.061	0.030	1.063	1.002–1.129	0.044
Model 2	0.061	0.031	1.063	1.001–1.129	0.046
Model 3	0.093	0.040	1.097	1.014–1.187	0.022

Model 1: adjusted for age, gender; Model 2: Model 1 + hypertension, diabetes, CHD; Model 3: Model 2 + WBC, cTnI, hs-CRP and Cr.

**Figure 1:**
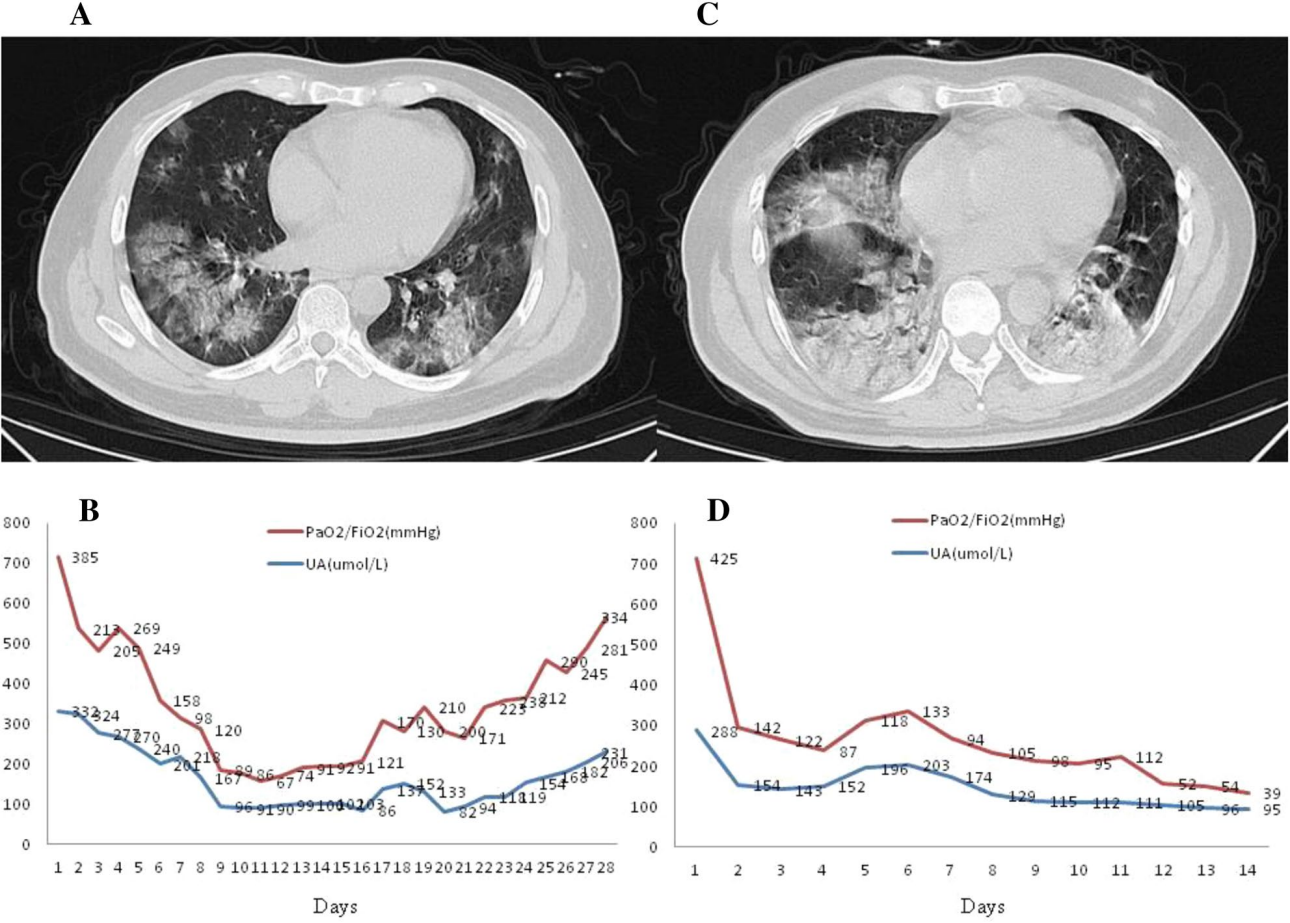
Chest CT images of COVID-19 patients and stacked trend chart of blood UA levels and PaO_2_/FiO_2_. (**A**) and (**B**) a 60 year old survivor’s CT images and stacked trend chart, the time point of the CT taken was the first day of admission (17th Jan, 2020); (**C**) and (**D**) a 51 year old non-survivor’s CT images and stacked trend chart, the time point of the CT taken was the first day of admission (15th Jan, 2020).

Figure 1 shows the computed tomography (CT) imaging results for two male COVID-19 patients upon admission to ICU and the change in uric acid and PaO_2_/FiO_2_ after admission. The left side (A, B) of Fig. 1 represents a CT image and trend chart for a 60-year-old male patient who survived. The right side (C, D) shows a CT image and trend chart for a 51-year-old male patient who eventually died. There was a significant decrease in uric acid level on the second day of admission in both dead and surviving patients. The correlation analysis indicated a significant correlation between the daily uric acid level and PaO_2_/FiO_2_ in these two patients(r = 0.689, *p* < 0.001).

**Figure 2:**
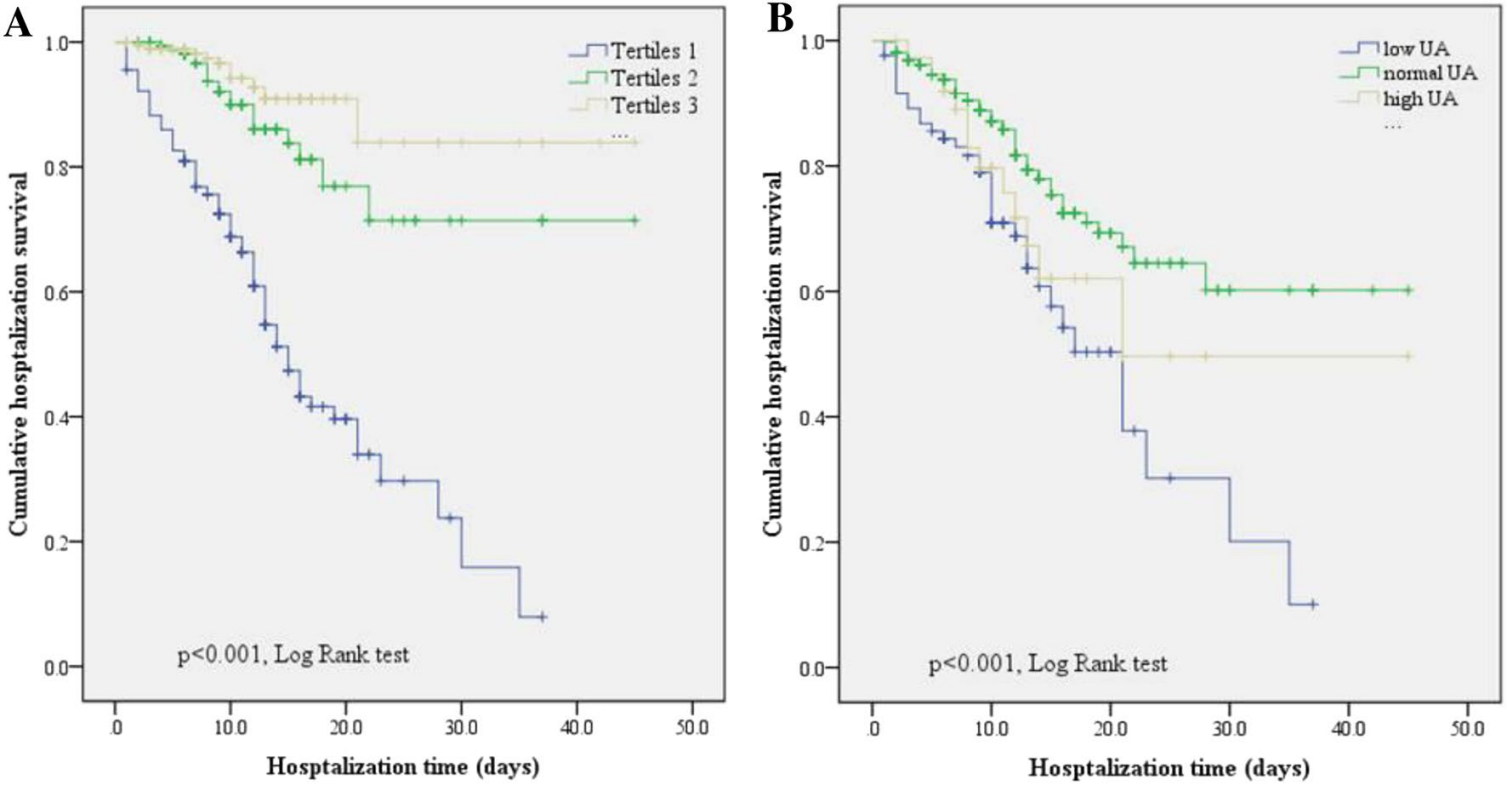
Kaplan–Meier curves stratified according to the lowest and admission UA level. Log-rank testing revealed a significant decrease between tertiles 1 group with other two groups (**A**). The mean survival time of the normal group was significantly higher than that in the low and high groups (**B**).

Patients were assigned into three groups according to the concentration of *low*UA: Tertile 1 (≤ 156 µmol/L), Tertile 2 (157–232 µmol/L), and Tertile 3 (≥ 223 µmol/L). Kaplan–Meier curve analysis showed that the mean survival time in the low-level group (Tertile 1: 17.4 days, 95% CI 15.0–19.8) was significantly lower than that in the Tertiles 2 (35.9 days, 95% CI 31.9–40.1, *p* < 0.001) and 3 groups (40.0 days, 95% CI 36.1–43.5, *p* < 0.001). However, there was no significant difference in the mean survival time between Tertiles 2 and 3 groups (Fig. 2A). According to the admission UA levels, we divided patients into low UA (≤ 154 µmol/L), normal UA (155–428 µmol/L) and high UA groups (≥ 429 µmol/L). Kaplan–Meier curve analysis showed that the mean survival time in the normal group (32.7 days, 95% CI 29.7–35.6, *p* < 0.001) was significantly higher than that in the low (19.3 days, 95% CI 15.7–23.0, *p* < 0.001) and high (28.5 days, 95% CI 20.8–36.2, *p* < 0.001) groups (Fig. 2B).

**Figure 3:**
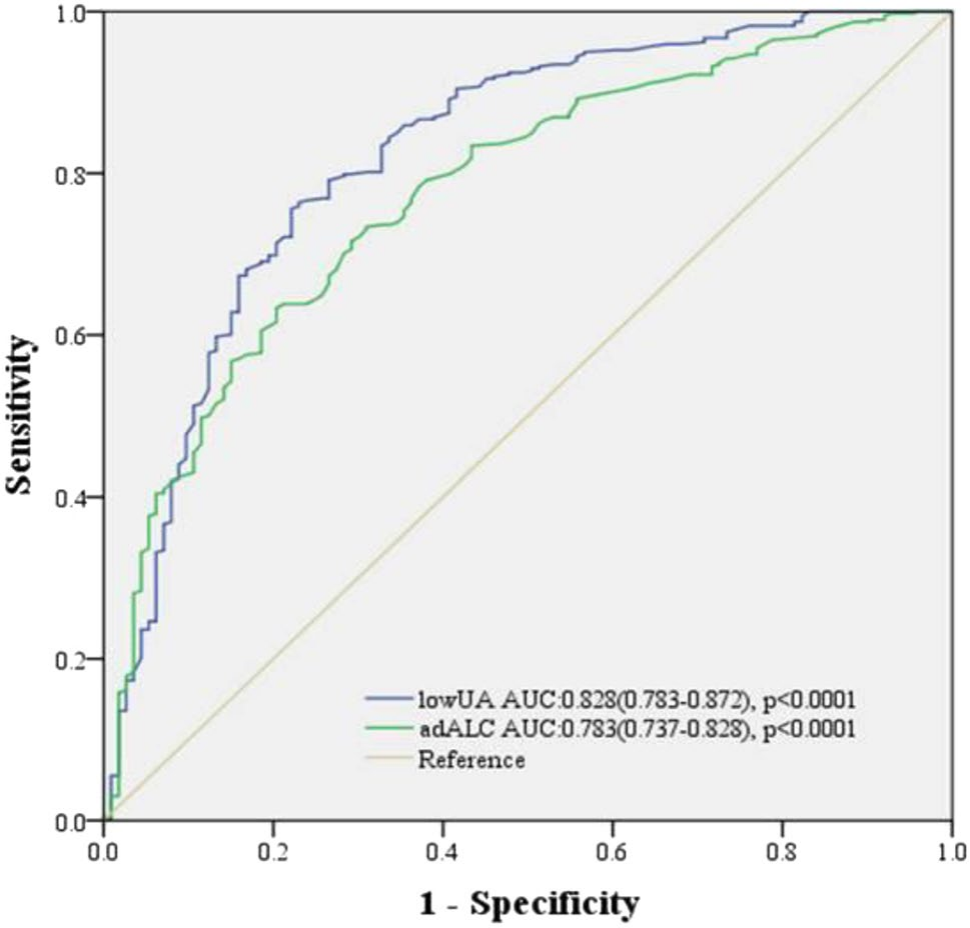
ROC curves showed the prognostic value of *low*UA and *ad*ALC in predicting in-hospital mortality.

Receiver operating characteristic (ROC) curve analysis was used to compare the predictive value of *low*UA and *ad*ALC for in-hospital death. The ROC curve showed that area under the curve (AUC) of the concentration of *low*UA was 0.828 (95% CI 0.783–0.872, *p* < 0.0001) and the cutoff value was 166 *u*mol/L (sensitivity: 76.9%, 95% CI 68.5–85.1%; specificity: 74.9% 95% CI 70.3–78.9%). The *ad*ALC AUC was 0.783 (95% CI 0.737–0.828, *p* < 0.0001) and the cutoff value was 0.94 (sensitivity: 79.3%, 95% CI 71.0–86.8%; specificity: 63.0% 95% CI 58.2–68.4%, Fig. 3).

## Discussion

This retrospective study of 540 patients with severe and critical COVID-19 found that low uric acid concentration was closely related to the risk of in-hospital death. The concentration of UA in the dead patients was significantly lower than that in the surviving patients. The lowest value of UA during hospitalization can independently predict the risk of in-hospital death. The mean survival time in the low UA group was significantly reduced.

Traditionally, high concentration of UA is closely related to hypertension, visceral obesity, insulin resistance, dyslipidemia, type II diabetes, kidney disease, and cardiovascular and cerebrovascular events^23,24^. However, UA is a natural product of purine catabolism that plays complex and variable roles in the body and is not just a metabolic end product. The metabolites of nucleic acids have been shown to have a significant effect on the natural immune system^25^. In the presence of nuclear factor-kappa B (NF-κB) signal, UA crystals can stimulate dendritic cells (DC) to promote the release of cytokines related to Th17 polarization, while UA can drive the differentiation of Th17 cells^26^. Allopurinol and uricase treatment can significantly reduce the concentration of plasma uric acid and inhibit T cell activation^27^. Studies in mice have shown that UA can increase T cell immune response and enhance its function by stimulating DC maturation. UA can also significantly enhance T cell immune response induced by HBsAg pulsed DCs vaccine^28^. In addition, UA can directly activate primary human T cells without antigen presentation^29^. Therefore, maintaining a certain level of UA is conducive to the activation of the immune system. The positive correlation between the UA concentration and lymphocyte count in the present study may verify this theory.

UA is often associated with inflammation and oxidative stress. However, UA also has certain antioxidant effects^17^. Experiments have shown that UA is a powerful scavenger for singlet oxygen, peroxyl radicals, and hydroxyl radicals^30^. High levels of urate circulation are considered to be one of the main antioxidants in plasma that protects cells from oxidative damage^17,31^. However, UA does not remove all free radicals, such as superoxide^32^. The ascorbic acid in the plasma is necessary for the antioxidation of UA^33^. Therefore, a large dose of ascorbic acid may enhance the antioxidant effect of UA. There was a negative correlation between the UA concentration and WBC and hs-CRP (reflecting inflammatory state) in severe and critically ill patients. Patients who died had lower UA levels and higher inflammatory levels. These suggest that low UA levels may indicate a higher inflammatory status and death risk in patients with COVID-19.

Sepsis often occurs in patients with COVID-19, especially in critical patients. A study from Tongji Hospital in Wuhan showed that sepsis occurred in 113 critically ill patients who eventually died^34^. In addition, 40% of the critical patients with COVID-19 who survived have sepsis. Zhou et al. found that blood and lower respiratory tract specimen cultures turned out to be negative for bacteria and fungus in 76% sepsis patients in a COVID-19 cohort^35^. COVID-19 may be the main cause of sepsis. We found that 80.0% of the dead patients had sepsis, and 48.9% of all patients had sepsis. Although there was no difference between the *ad*UA of patients with sepsis and that of patients without sepsis, the *low*UA of patients with sepsis is significantly lower than that of patients without sepsis. In the Chen study, the median PCT level was 0.33 ng/mL in patients who died and 0.09 ng/mL in patients who survived^34^. The level of PCT in dead patients with COVID-19 was significantly lower than that in other patients with bacterial infection. SARS-CoV-2 could directly infect lymphocytes, particularly T cells, and initiate or promote the cell death of lymphocytes, which eventually lead to lymphopenia and impaired antiviral responses^36^. It has been reported that hyperuricemia can reflect the early severity of sepsis^37^. However, in these studies, the cause of sepsis was mostly bacterial infection, not viral sepsis. The mechanism of uric acid in viral sepsis needs more research to further confirm.

A significant decrease in UA levels was present in patients after admission. UA levels in the dead patients decreased at a greater rate in a shorter time period. The mean survival time of patients in the Tertile 1 group was significantly lower than that in the other two groups. UA levels dropping to 163 µmol/L was a dangerous signal. When the change in UA levels was observed in some severe patients with COVID-19 during hospitalization, it was found that UA fluctuated slightly for 3–5 days near the lowest level. The lowest level of UA in the survivors rose rapidly after a short period of fluctuation, while that in the soon-to-be dead patients continued to fluctuate until death. Therefore, the change in UA can help to judge the turning point of patients' condition. In a retrospective study using two independent cohorts, among patients with COVID-19 requiring hospitalization, low serum levels of uric acid are common and associate with disease severity and with progression to respiratory failure requiring invasive mechanical ventilation^38^. However, a study cohort included 1854 patients showed that the association between admission serum uric acid and composite outcome of COVID-19 patients was U-shaped. They found that compared with baseline serum uric acid levels of 279–422 μmol/L, values ≥ 423 μmol/L were associated with an increased risk of composite outcome and mechanical ventilation, whereas levels ≤ 278 μmol/L associated with increased risk of composite outcome, ICU admission and mechanical ventilation^39^. In the present study, we also found that high or low admission UA levels will reduce the survival time of patients. The relationship between serum uric acid and prognosis of COVID-19 patients is complicated and it is need more study to clarify in the future.

Glucocorticoids were important steroid hormones. Many clinical trials have tested the utility of corticosteroids in critically ill patients with pneumonia, septic shock, or ARDS. Over the past 3 years, well conducted randomized clinical trials (RCTs) have suggested benefit of corticosteroids in ARDS and septic shock. The DEXA-ARDS trial enrolled 277 patients with moderate to severe ARDS and found that patients randomized to high-dose dexamethasone compared with continued routine intensive care had lower 60-day all cause mortality and more ventilator free days^40^. In the UK-based RECOVERY trial, a large open-label randomized trial enrolling 6425 patients, treatment with dexamethasone reduced mortality by one-third in patients receiving mechanical ventilation and by one-fifth in patients receiving supplemental oxygen compared with usual care alone^41^. However, a meta-analysis of 21,350 patients with COVID-19 concluded that overall mortality was greater among patients with the disease who were receiving corticosteroids than among patients who were not treated with corticosteroids^42^. Corticosteroids as an inexpensive drug provide some evidence and hope during the pandemic^43^. Whether corticosteroids can affect the change of blood uric acid level in patients with COVID-19 still needs further study.

### Limitations

There are some shortcomings in the present study. First, only patients with severe and critical diseases were included and UA changes in patients with ordinary infections were not observed. However, 80% of the infected patients were mild patients. Second, patient viral load was not tested based on a theoretical speculation that UA can reflect the virus activity in patients. More prospective studies are needed in the future to evaluate this concern. Third, this cohort is a derivation cohort but not a validation cohort. The real strength of UA as a prognostic marker would only be confirmed when the optimized cutoff value from this cohort for *low*UA is tested and confirmed in a separate validation cohort. Finally, due to the centralized outbreak of COVID-19, it is difficult to receive and treat patients. It is likely that a considerable number of patients have been in a state of reduced UA upon admission, which may lead to some bias during statistical analysis.

## Conclusions

In conclusion, this retrospective study determined that UA, a purine base metabolite, can be used as a prognostic indicator in severe patients with COVID-19. In the future, whether UA can accurately reflect the viral load still needs to be investigated.

## Data Availability

All data generated or analyzed in this study are included in this published article, and the datasets are available from the corresponding author within the limits imposed by ethical and legal dispositions.
